# Reduction Theorem for Secrecy over Linear Network Code for Active Attacks †

**DOI:** 10.3390/e22091053

**Published:** 2020-09-21

**Authors:** Masahito Hayashi, Masaki Owari, Go Kato, Ning Cai

**Affiliations:** 1 Shenzhen Institute for Quantum Science and Engineering, Southern University of Science and Technology, Shenzhen 518055, China; 2 Guangdong Provincial Key Laboratory of Quantum Science and Engineering, Southern University of Science and Technology, Shenzhen 518055, China; 3 Shenzhen Key Laboratory of Quantum Science and Engineering, Southern University of Science and Technology, Shenzhen 518055, China; 4 Graduate School of Mathematics, Nagoya University, Nagoya 464-8602, Japan; 5 Department of Computer Science, Faculty of Informatics, Shizuoka University, Shizuoka 422-8529, Japan; masakiowari@inf.shizuoka.ac.jp; 6 NTT Communication Science Laboratories, NTT Corporation, Tokyo 100-8116, Japan; go.kato.gm@hco.ntt.co.jp; 7 The School of Information Science and Technology, ShanghaiTech University, Middle Huaxia Road no. 393, Pudong, Shanghai 201210, China; ningcai@shanghaitech.edu.cn

**Keywords:** secrecy analysis, secure network coding, sequential injection, passive attack, active attack

## Abstract

We discuss the effect of sequential error injection on information leakage under a network code. We formulate a network code for the single transmission setting and the multiple transmission setting. Under this formulation, we show that the eavesdropper cannot increase the power of eavesdropping by sequential error injection when the operations in the network are linear operations. We demonstrated the usefulness of this reduction theorem by applying a concrete example of network.

## 1. Introduction

Secure network coding offers a method for securely transmitting information from an authorized sender to an authorized receiver. Cai and Yeung [1] discussed the secrecy of when a malicious adversary, Eve, wiretaps a subset $E_{E}$ of the set *E* of all the channels in a network. Using the universal hashing lemma [2,3,4], the papers [5,6] showed the existence of a secrecy code that works universally for any type of eavesdropper when the cardinality of $E_{E}$ is bounded. In addition, the paper [7] discussed the construction of such a code. As another type of attack on information transmission via a network, a malicious adversary contaminates the communication by changing the information on a subset $E_{A}$ of *E*. Using an error correcting code, the papers [8,9,10,11] proposed a method to protect the message from contamination. That is, we require that the authorized receiver correctly recovers the message, which is called robustness.

As another possibility, we consider the case when the malicious adversary combines eavesdropping and contamination. That is, by contaminating a part of the channels in the network, the malicious adversary might increase the ability of eavesdropping, whereas a parallel network offers no such a possibility [12,13,14]. In fact, in arbitrarily varying channel model, noise injection is allowed after Eve’s eavesdropping, but Eve does not eavesdrop the channel after Eve’s noise injection [15,16,17,18,19]. The paper [20] also discusses secrecy in the same setting while it addresses the network model. The studies [7,14] discussed the secrecy when Eve eavesdrops the information transmitted on the channels in $E_{E}$ after noises are injected in $E_{A}$, but they assume that Eve does not know the information of the injected noise.

In contrast, this paper focuses on a network, and discusses the secrecy when Eve adds artificial information to the information transmitted on the channels in $E_{A}$, eavesdrops on the information transmitted on the channels in $E_{E}$, and estimates the original message from the eavesdropped information and the information of the injected noises. We call this type of attack an active attack and call an attack without contamination a passive attack. We call each of Eve’s active operations a strategy. When $E_{A}\subset E_{E}$ and any active attack are available for Eve, she is allowed to arbitrarily modify the information on the channels in $E_{A}$ sequentially based on the obtained information.

This paper aims to show a reduction theorem for an active attack, i.e., the fact that no strategy can increase Eve’s information when every operation in the network is linear and Eve’s contamination satisfies a natural causal condition. When the network is not well synchronized, Eve can make an attack across several channels. This reduction theorem holds even under this kind of attack. In fact, there is an example having a non-linear node operation such that Eve can increase her performance to extract information from eavesdropping an edge outgoing an intermediate node by adding artificial information to an edge incoming the intermediate node [21] (The paper [21] also discusses linear code; it discusses only code a on one-hop relay network. Our results can be applied to general networks.) This example shows the necessity of linearity for this reduction theorem. Although our discussion can be extended to the multicast and multiple-unicast cases, for simplicity, we consider the unicast setting in the following discussion.

Further, we apply our general result to the analysis of a concrete example of a network. In this network, we demonstrate that any active attack cannot increase the performance of eavesdropping. However, in the single transmission case over the finite field $F_{2}$, the error correction and the error detection are impossible over this contamination. To resolve this problem, this paper addresses the multiple transmission case in addition to the single transmission case. In the multiple transmission case, the sender uses the same network multiple times, and the topology and dynamics of the network do not change during these transmissions. While several papers discussed this model, many of them discussed the multiple transmission case only with contamination [22,23,24] or eavesdropping [5,6,25,26,27]. Only the paper [20] addressed it with contamination and eavesdropping, and its distinction from the paper [20] is summarized as follows. The paper [20] assumes that all injections are done after eavesdropping, while this paper allows Eve to inject the artificial information before a part of eavesdropping.

We formulate the multiple transmission case when each transmission has no correlation with the previous transmission while injected noise might have such a correlation. Then, we show the above type of reduction theorem for an active attack even under the multiple transmission case. We apply this result to the multiple transmission over the above example of a network, in which the error correction and the error detection are possible over this contamination. Hence, the secrecy and the correctness hold in this case.

The remaining part of this paper is organized as follows. Section 2 discusses the single transmission setting that has only a single transmission, and Section 3 discusses the multiple transmission setting that has *n* transmissions. Two types of multiple transmission setting are formulated. Then, we state our reduction theorem in both settings. In Section 4, we state the conclusion.

## 2. Single Transmission Setting

### 2.1. Generic Model

In this subsection, we give a generic model, and discuss its relation with a concrete network model in the latter subsections. We consider the unicast setting of network coding on a network. Assume that the authorized sender, Alice, intends to send information to the authorized receiver, Bob, via the network. Although the network is composed of $m_{1}$ edges and $m_{2}$ vertecies, as shown later, the model can be simplified as follows when the node operations are linear. We assume that Alice inputs the input variable X in $F_{q}^{m_{3}}$ and Bob receives the output variable $Y_{B}$ in $F_{q}^{m_{4}}$, where $F_{q}$ is a finite field whose order is a power *q* of a prime *p*. We also assume that the malicious adversary, Eve, wiretaps the information $Y_{E}$ in $F_{q}^{m_{6}}$ This network has $m_{7}=m_{1}-m_{3}$ edges that are not directly linked to the source node. The parameters are summarized as Table 1. (In this paper, we denote the vector on $F_{q}$ by a bold letter, but we use a non-bold letter to describe a scalar and a matrix).

Then, we adopt the model with matrices $K_{B}=\left[K_{B;j,i}\right]\in F_{q}^{m_{4}\times m_{3}}$ and $K_{E}=\left[K_{E;j,i}\right]\in F_{q}^{m_{6}\times m_{3}}$, in which the variables X, $Y_{B}$, and $Y_{E}$ satisfy their relations

(1) $$\begin{array}{c} Y_{B}=K_{B}X,\;Y_{E}=K_{E}X. \end{array}$$

This attack is a conventional wiretap model and is called a passive attack to distinguish it from an active attack, which will be introduced later. Section 2.3 will explain how this model is derived from a directed graph with $E_{E}$ and linear operations on nodes.

In this paper, we address a stronger attack, in which Eve injects noise $Z\in F_{q}^{m_{5}}$. Hence, using matrices $H_{B}=\left[H_{B;j,i}\right]\in F_{q}^{m_{4}\times m_{5}}$ and $H_{E}=\left[H_{E;j,i}\right]\in F_{q}^{m_{6}\times m_{5}}$, we rewrite the relations (1) as

(2) $$\begin{array}{c} Y_{B}=K_{B}X+H_{B}Z,\;Y_{E}=K_{E}X+H_{E}Z, \end{array}$$

which is called a wiretap and addition model. The *i*-th injected noise $Z_{i}$ (the *i*-th component of Z) is decided by a function $\alpha_{i}$ of $Y_{E}$. Although a part of $Y_{E}$ is a function of $\alpha_{i}$, this point does not make a problem for causality, as explained in Section 2.5. In this paper, when a vector has the *j*-th component $x_{j}$; the vector is written as ${\left[x_{j}\right]}_{1\leq j\leq a}$, where the subscript 1≤j≤a expresses the range of the index *j*. Thus, the set $\alpha ={\left[\alpha_{i}\right]}_{1\leq i\leq m_{5}}$ of the functions can be regarded as Eve’s strategy, and we call this attack an active attack with a strategy α. That is, an active attack is identified by a pair of a strategy α and a wiretap, and an addition model decided by K,H. Here, we treat $K_{B},K_{E},H_{B}$, and $H_{E}$ as deterministic values, and denote the pairs $\left(K_{B},K_{E}\right)$ and $\left(H_{B},H_{E}\right)$ by K and H, respectively. Hence, our model is written as the triplet (K,H,α). As shown in the latter subsections, under the linearity assumption on the node operations, the triplet (K,H,α) is decided from the network topology (a directed graph with $E_{A}$ and $E_{E}$) and dynamics of the network. Here, we should remark that the relation (2) is based on the linearity assumption for node operations. Since this assumption is the restriction for the protocol, it does not restrict the eavesdropper’s strategy.

We impose several types for regularity conditions for Eve’s strategy α, which are demanded from causality. Notice that $\alpha_{i}$ is a function of the vector ${\left[Y_{E,j}\right]}_{1\leq j\leq m_{6}}$. Now, we take the causality with respect to α into account. Here, we assume that the assigned index *i* for $1\leq i\leq m_{5}$ expresses the time-ordering of injection. That is, we assign the index *i* for $1\leq i\leq m_{5}$ according to the order of injections. Hence, we assume that $\alpha_{i}$ is decided by a part of Eve’s observed variables. We say that subsets $w_{i}\subset \left\{1,\ldots ,m_{6}\right\}$ for $i\in \{1,\ldots ,m_{5}\}$ are the domain index subsets for α when the function $\alpha_{i}$ is given as a function of the vector ${\left[Y_{E,j}\right]}_{j\in w_{i}}$. Here, the notation $j\in w_{i}$ means that the *j*-th eavesdropping is done before the *i*-th injection; i.e., $w_{i}$ expresses the set of indexes corresponding to the symbols that do effect the *i*-th injection. Hence, the eavesdropped symbol $Y_{E,j}$ does not depend on the injected symbol $z_{i}$ for $j\in w_{i}$. Since the decision of the injected noise does not depend on the consequences of the decision, we introduce the following causal condition.

**Definition** **1.** *We say that the domain index subsets ${\left\{w_{i}\right\}}_{1,\ldots ,m_{5}}$ satisfy the causal condition when the following two conditions hold:*

**(A1)**  *The relation $H_{E;j,i}=0$ holds for $j\in w_{i}$.*
**(A2)**  *The relation $w_{1}\subseteq w_{2}\subseteq \ldots \subseteq w_{m_{5}}$ holds.*

As a necessary condition of the causal condition, we introduce the following uniqueness condition for the function $\alpha_{i}$, which is given as a function of the vector ${\left[Y_{E,j}\right]}_{1\leq j\leq m_{6}}$.

**Definition** **2.** *For any value of x, there uniquely exists $y\in F_{q}^{m_{6}}$ such that*

(3) $$\begin{array}{c} y=K_{E}x+H_{E}\alpha \left(y\right). \end{array}$$

*This condition is called the uniqueness condition for α.*

When the uniqueness condition does not hold, for an input x, there exist two vectors y and $y^{\prime }$ to satisfy (3). It means that both outputs y and $y^{\prime }$ may happen; nevertheless, all the operations are deterministic. This situation is unlikely in a real word. Examples of a network with $w_{i}$, ${\left[H_{E;j,i}\right]}_{i,j}$ will be given in Section 2.6. Then, we have the following lemma, which shows that the uniqueness condition always holds under a realistic situation.

**Lemma** **1.** *When a strategy α has domain index subsets to satisfy the causal condition, the strategy α satisfies the uniqueness condition.*

**Proof.**  When the causal condition holds, we show the fact that $y_{j^{\prime }}$ is given as a function of $K_{E}x$ for any $j^{\prime }\in w_{i}$ by induction with respect to the index $i=1,\ldots ,m_{5}$, which expresses the order of the injected information. This fact yields the uniqueness condition.

For $j\in w_{1}$, we have $y_{j}={\left(K_{E}x\right)}_{j}$ because ${\left(H_{E}\alpha \left(y\right)\right)}_{j}$ is zero. Hence, the statement with i=1 holds. We choose $j\in w_{i+1}\setminus w_{i}$. Let $z_{i^{\prime }}$ be the $i^{\prime }$-th injected information. Due to conditions (A1) and (A2), $y_{j}-{\left(K_{E}x\right)}_{j}={\left(H_{E}z\right)}_{j}$ is a function of $z_{1}=\alpha {\left(y\right)}_{1},\cdots ,z_{i}=\alpha {\left(y\right)}_{i}$. Since the assumption of the induction guarantees that $z_{1},\ldots ,z_{i}$ are functions of ${\left[y_{j^{\prime }}\right]}_{j^{\prime }\in w_{i}}$, $z_{1},\ldots ,z_{i}$ are functions of $K_{E}x$. Then, we find that $y_{j}={\left(K_{E}x\right)}_{j}+{\left(H_{E}z\right)}_{j}$ is given as a function of $K_{E}x$ for any $j\in w_{i+1}\setminus w_{i}$. That is, the strategy α satisfies the uniqueness condition. □

Now, we have the following reduction theorem.

**Theorem** **1** (Reduction theorem)**.** *When the strategy α satisfies the uniqueness condition, Eve’s information $Y_{E}\left(\alpha \right)$ with strategy α can be calculated from Eve’s information $Y_{E}\left(0\right)$ with strategy 0 (the passive attack), and $Y_{E}\left(0\right)$ is also calculated from $Y_{E}\left(\alpha \right)$. Hence, we have the equation*

(4) $$\begin{array}{cc} I\left(X;Y_{E}\right)\left[0\right]= & I\left(X;Y_{E}\right)\left[\alpha \right], \end{array}$$

*$I\left(X;Y_{E}\right)\left[\alpha \right]$ expresses the mutual information between X and $Y_{E}$ under the strategy α.*

**Proof.**  This proof can be done by showing that Eve’s information with a strategy α can be simulated by Eve’s information with a strategy 0 as follows.

Since $Y_{E}\left(0\right)=K_{E}X$ and $Y_{E}\left(\alpha \right)=K_{E}X+H_{E}Z$, due to the uniqueness condition of the strategy α, we can uniquely evaluate $Y_{E}\left(\alpha \right)$ from $Y_{E}\left(0\right)=K_{E}X$ and α. Therefore, we have $I\left(X;Y_{E}\right)\left[0\right]\geq I\left(X;Y_{E}\right)\left[\alpha \right]$. Conversely, since $Y_{E}\left(0\right)$ is given as a function ($Y_{E}\left(\alpha \right)-H_{E}Z$) of $Y_{E}\left(\alpha \right)$, Z, and $H_{E}$, we have the opposite inequality. □

This theorem shows that the information leakage of the active attack with the strategy α is the same as the information leakage of the passive attack. Hence, to guarantee the secrecy under an arbitrary active attack, it is sufficient to show secrecy under the passive attack. However, there is an example of non-linear network such that this kind of reduction does not hold [21]. In fact, even when the network does not have synchronization so that the information transmission on an edges starts before the end of the information transmission on the previous edge, the above reduction theorem hold under the uniqueness condition.

### 2.2. Recovery and Information Leakage with Linear Code

Next, we consider the recovery and the information leakage when a linear code is used. Assume that a message $M\in F_{q}^{\ell }$ is generated subject to the uniform distribution and is sent via an encoding map, i.e., a linear map $f_{1}$ from $F_{q}^{\ell }$ to $F_{q}^{m_{3}}$. Additionally, Alice independently generates a scramble variable $L\in F_{q}^{k}$ subject to the uniform distribution and send it via another a linear map $f_{2}$ from $F_{q}^{k}$ to $F_{q}^{m_{3}}$. In this case, Alice transmits $f_{1}\left(M\right)+f_{2}\left(L\right)\in F_{q}^{m_{3}}$, as is implicitly stated in many papers ([22] Section V; [23] Section V; [20] Section IV).

**Proposition** **1.** *Bob is assumed to know the forms of $K_{B},H_{B}$ and $f_{1}$, $f_{2}$. Bob can correctly recover the message M with probability 1 if and only if dimImKB∘f1=ℓ and dim(ImKB∘f1∩(ImKB∘f2+ImHB))=0.*

**Proof.**  To recover the message *M*, the dimension dimImKB∘f1 needs to be *ℓ*. When dim(ImKB∘f1∩(ImKB∘f2+ImHB))>0, there exist vectors $m_{0}\left(\neq 0\right)\in F_{q}^{\ell }$, $l_{0}\in F_{q}^{k}$, and $z_{0}\in F_{q}^{m_{5}}$ such that $K_{B}\left(f_{1}\left(m_{0}\right)+f_{2}\left(l_{0}\right)\right)+H_{B}\left(z_{0}\right)=0$. Thus, Bob may receive 0 when the message M is 0 or $m_{0}$. This fact means the impossibility of Bob’s perfect recovery.

When dimImKB∘f1=ℓ and dim(ImKB∘f1∩(ImKB∘f2+ImHB))=0, there exists a linear map *P* from $F_{q}^{m_{4}}$ to ImKB∘f1 such that Pu=u for u∈ImKB∘f1 and $Pu^{\prime }=0$ for u′∈ImKB∘f2+ImHB. By applying the map *P*, Bob recovers the message M. □

Assume that Bob knows $K_{B}$ but does not know the form of $H_{B}$; i.e., there are several possible forms $H_{B,1},\ldots ,H_{B,d}$ as the candidate of $H_{B}$. Additionally, we assume that all possible forms $H_{B,1},\ldots ,H_{B,d}$ satisfy the condition of Proposition 1. In general, the map *P* used in the proof depends on the form of $H_{B}$. When the map *P* can be chosen commonly with $H_{B,1},\ldots ,H_{B,d}$, Bob can recover the message M. Otherwise, Bob cannot recover it.

However, when the condition dim(ImKB∘f2∩ImHB,i)=0 holds in addition to the condition of Proposition 1 for i=1,…,d, Bob can detect the existence of contamination as follows. In this case, when $Y_{B}$ does not belong to ImKB∘f1+ImKB∘f2, Bob considers that a contamination exists. In other words, when we choose a linear function $f_{3}$ such that {yB∈Fqm3|f3(yB)=0}=ImKB∘f1+ImKB∘f2, the existence of a contamination can be detected by checking the condition $f_{3}\left(Y_{B}\right)=0$.

When the strategy α satisfies the uniqueness condition, Eve’s recovery can be reduced to the case with Z=0 due to Theorem 1. Therefore, Eve can correctly recover the message M if and only if dimImKE∘f1=ℓ and dim(ImKE∘f1∩ImKE∘f2)=0.

For the amount of information leakage, the papers [28] (Theorem 2), and [29] (Corollary 3.3 and (25)) stated the following relation in a slightly different way.

**Proposition** **2.** *Information leakage to Eve can be evaluated as I(M;YE)[0]=(logq)(dimImKE∘f1−dim(ImKE∘f1∩ImKE∘f2)). In particular, $I\left(M;Y_{E}\right)\left[0\right]=0$ if and only if dimImKE∘f1=dim(ImKE∘f1∩ImKE∘f2).*

**Proof.**  Consider the case with α=0. H(YE)=(logq)dim(ImKE∘f1+ImKE∘f2) and H(YE|M)=(logq)dimImKE∘f2. Hence, I(M;YE)[0]=(logq)dim(ImKE∘f1+ImKE∘f2)−(logq)dimImKE∘f2=(logq)(dimImKE∘f1−dim(ImKE∘f1∩ImKE∘f2)). □

Therefore, using Proposition 2, we can evaluate the amount of leaked information even for general strategy α.

To check the condition dimImKE∘f1=dim(ImKE∘f1∩ImKE∘f2), we introduce two matrices $A_{1}\in F_{q}^{m_{6}\times \ell }$ and $A_{2}\in F_{q}^{m_{6}\times k}$ by $K_{E}\circ f_{1}\left(m\right)=A_{1}m$ for $m\in F_{q}^{\ell }$ and $K_{E}\circ f_{2}\left(l\right)=A_{2}l$ for $l\in F_{q}^{k}$. Then, we define $m_{6}$ low vectors $v_{i}$ for $i=1,\ldots ,m_{6}$ of the matrix $A:=(A_{1}A_{2})$. Considering an equivalent condition to dimImKE∘f1=dim(ImKE∘f1∩ImKE∘f2), we have the following corollary.

**Corollary** **1.** *$I\left(M;Y_{E}\right)\left[0\right]=0$ if and only if there does not exist a vector $\left(b_{1},\ldots ,b_{m_{6}}\right)\in F_{q}^{m_{6}}$ such that $\sum_{i=1}^{m_{6}}b_{i}v_{i}$ has a form $\left(m,\underset{k}{\underset{︸}{0,\ldots ,0}}\right)$ with $m\neq 0\in F_{q}^{\ell }$.*

### 2.3. Construction of $K_{B},K_{E}$ from Concrete Network Model

Next, we discuss how we can obtain the generic passive attack model (1) from a concretely structured network coding, i.e., communications identified by directed edges and linear operations by parties identified by nodes. We consider the unicast setting of network coding on a network, which is given as a directed graph (V,E), where the set $V:=\{v\left(1\right),\ldots ,v\left(m_{2}\right)\}$ of vertices expresses the set of nodes and the set $E:=\{e\left(1\right),\ldots ,e\left(m_{1}\right)\}$ of edges expresses the set of communication channels, where a communication channel means a packet in network engineering; i.e., a single communication channel can transmit single character in $F_{q}$. In the following, we identify the set *E* with $\left\{1,\ldots ,m_{1}\right\}$; i.e, we identify the index of an edge with the edge itself. Here, the directed graph (V,E) is not necessarily acyclic. When a channel transmits information from a node v(i)∈V to another node $v(i^{\prime })\in V$, it is written as $(v\left(i\right),v\left(i^{\prime }\right))\in E$.

In the single transmission, the source node has several elements of $F_{q}$ and sends each of them via its outgoing edges in the order of assigned number of edges. Each intermediate node keeps received information via incoming edges. Then, for each outgoing edge, the intermediate node calculates one element of $F_{q}$ from previously received information, and sends it via the outgoing edge. That is, every outgoing piece of information from a node v(i) via a channel e(j) depends only on the information coming into the node v(i) via channels $e(j^{\prime })$ such that $j^{\prime }<j$. The operations on all nodes are assumed to be linear on the finite field $F_{q}$ with prime power *q*. Bob receives the information $Y_{B}$ in $F_{q}^{m_{4}}$ on the edges of a subset $E_{B}:=\left\{e\left(\zeta_{B}\left(1\right)\right),\ldots ,e\left(\zeta_{B}\left(m_{4}\right)\right)\right\}\subset E$, where $\zeta_{B}$ is a strictly increasing function from $\left\{1,\ldots ,m_{4}\right\}$ to $\left\{1,\ldots ,m_{1}\right\}$. Let ${\tilde{X}}_{j}$ be the information on the edge e(j). In the following, we describe the information on the $m_{7}=m_{1}-m_{3}$ edges that are not directly linked to the source node because $m_{3}$ expresses the number of Alice’s input symbols. When the edge e(j) is an outgoing edge of the node v(i), the information ${\tilde{X}}_{j}$ is given as a linear combination of the information on the edges coming into the node v(i). We chose an $m_{1}\times m_{1}$ matrix $\theta =(\theta_{j,j^{\prime }})$ such that ${\tilde{X}}_{j}=\sum_{j^{\prime }}\theta_{j,j^{\prime }}{\tilde{X}}_{j^{\prime }}$, where $\theta_{j,j^{\prime }}$ is zero unless $e(j^{\prime })$ is an edge incoming to v(i). The matrix θ is the coefficient matrix of this network.

Now, from causality, we can assume that each node makes the transmissions on the outgoing edges in the order of the numbers assigned to the edges. At the first stage, all $m_{3}$ information generated at the source node is directly transmitted via $e\left(1\right),\cdots e\left(m_{3}\right)$ respectively. Then, at time *j*, the information transmission on the edge $e(j+m_{3})$ is done. Hence, naturally, we impose the condition

(5) $$\begin{array}{c} \theta_{j,j^{\prime }}=0\;\mathrm{for}\;j^{\prime }\geq j, \end{array}$$

which is called the partial time-ordered condition for θ. Then, to describe the information on $m_{7}$ edges that is not directly linked to the source node, we define $m_{7}$ $m_{1}\times m_{1}$ matrices $M_{1},\ldots ,M_{m_{7}}$. The *j*-th $m_{1}\times m_{1}$ matrix $M_{j}$ gives the information on the edge $e(j+m_{3})$ as a function of the information on edges ${\left\{e\left(j^{\prime }\right)\right\}}_{1\leq j^{\prime }\leq m_{1}}$ at time *j*. The $j+m_{3}$-th row vector of the matrix $M_{j}$ is defined by ${\left[\theta_{j+m_{3},j^{\prime }}\right]}_{1\leq j^{\prime }\leq m_{1}}$. The remaining part of $M_{j}$, i.e., the *i*-th row vector for $i\neq j+m_{3}$, is defined by ${\left[\delta_{i,j^{\prime }}\right]}_{1\leq j^{\prime }\leq m_{1}}$ and $\delta_{i,j^{\prime }}$ is the Kronecker delta. Since $\sum_{i=1}^{m_{3}}{\left(M_{j}\cdots M_{1}\right)}_{j^{\prime },i}X_{i}$ expresses the information on edge $e(j^{\prime })$ at time *j*, we have

(6) $$\begin{array}{c} Y_{B,j}=\sum_{i=1}^{m_{3}}{\left(M_{m_{7}}\cdots M_{1}\right)}_{\zeta_{B}\left(j\right),i}X_{i}. \end{array}$$

While the output of the matrix $M_{m_{7}}\cdots M_{1}$ takes values in $F_{q}^{m_{1}}$, we focus the projection $P_{B}$ to the subspace $F_{q}^{m_{4}}$ that corresponds to the $m_{4}$ components observed by Bob. That is, $P_{B}$ is a $m_{4}\times m_{1}$ matrix to satisfy $P_{B;i,j}=\delta_{\zeta_{B}\left(i\right),j}$. Similarly, we use the projection $P_{A}$ (an $m_{1}\times m_{3}$ matrix) as $P_{A;i,j}=\delta_{i,j}$. Due to (6), the matrix $K_{B}:=P_{B}M_{m_{7}}\cdots M_{1}P_{A}$ satisfies the first equation in (1).

The malicious adversary, Eve, wiretaps the information $Y_{E}$ in $F_{q}^{m_{6}}$ on the edges of a subset $E_{E}:=\left\{e\left(\zeta_{E}\left(1\right)\right),\ldots ,e\left(\zeta_{E}\left(m_{6}\right)\right)\right\}\subset E$, where $\zeta_{E}$ is a strictly increasing function from $\left\{1,\ldots ,m_{6}\right\}$ to $\left\{1,\ldots ,m_{1}\right\}$. Similar to (6), we have

(7) $$\begin{array}{c} Y_{E,j}=\sum_{i=1}^{m_{3}}{\left(M_{m_{7}}\cdots M_{1}\right)}_{\zeta_{E}\left(j\right),i}X_{i}. \end{array}$$

We employ the projection $P_{E}$ (an $m_{6}\times m_{1}$ matrix) to the subspace $F_{q}^{m_{6}}$ that corresponds to the $m_{6}$ components eavesdropped by Eve. That is, $P_{E;i,j}=\delta_{\zeta_{E}\left(i\right),j}$. Then, we obtain the matrix $K_{E}$ as $P_{E}M_{m_{7}}\cdots M_{1}P_{A}$. Due to (6), the matrix $K_{E}:=P_{E}M_{m_{7}}\cdots M_{1}P_{A}$ satisfies the second equation in (1).

In summary the topology and dynamics (operations on the intermediate nodes) of the network, including the places of attached edges decides the graph (V,E), the coefficients $\theta_{i,j}$, and functions $\zeta_{B},\zeta_{E}$, uniquely give the two matrices $K_{B}$ and $K_{E}$. Section 2.6 gives an example for this model. Here, we emphasize that we do not assume the acyclic condition for the graph (V,E). We can use this relaxed condition because we have only one transmission in the current discussion. That is, due to the partial time-ordered condition for θ, we can uniquely define our matrices $K_{B}$ and $K_{E}$, in a similar way to [30] (Section V-B; Λ of Ahlswede–Cai–Li–Yeung corresponds to the number of edges that are not connected to the source node in our paper.) However, when the graph has a cycle and we have *n* transmissions, there is a possibility of a correlation with the delayed information that is dependent on the time ordering. As a result, it is difficult to analyze secrecy for the cyclic network coding.

### 2.4. Construction of $H_{B},H_{E}$ from a Concrete Network Model

We identify the wiretap and addition model from a concrete network structure. We assume that Eve injects the noise in a part of edges $E_{A}\subset E$ and eavesdrops the edges $E_{E}$.

The elements of the subset $E_{A}$ are expressed as $E_{A}=\left\{e\left(\eta \left(1\right)\right),\ldots ,e\left(\eta \left(m_{5}\right)\right)\right\}$ by using a function η from $\left\{1,\ldots ,m_{5}\right\}$ to $\left\{1,\ldots ,m_{1}\right\}$, where the function η is not necessarily monotonically increasing function. To give the matrices $H_{B}$ and $H_{E}$, while modifying the matrix $M_{j}$, we define the new matrix $M_{j}^{\prime }$ as follows The $j+m_{3}$-th row vector of the new matrix $M_{j}^{\prime }$ is defined by ${\left[\theta_{j+m_{3},j^{\prime }}+\delta_{j+m_{3},j^{\prime }}\right]}_{1\leq j^{\prime }\leq m_{1}}$. The remaining part of $M_{j}^{\prime }$, i.e., the *i*-th row vector for $i\neq j+m_{3}$, is defined by ${\left[\delta_{i,j^{\prime }}\right]}_{1\leq j^{\prime }\leq m_{1}}$. Since $\sum_{i=1}^{m_{3}}{\left(M_{j}\cdots M_{1}\right)}_{j^{\prime },i}X_{i}+\sum_{i^{\prime }=1}^{m_{5}}{\left(M_{j}^{\prime }\cdots M_{1}^{\prime }\right)}_{j^{\prime },\eta \left(i^{\prime }\right)}Z_{i^{\prime }}$ expresses the information on edge $e(j^{\prime })$ at time *j*, we have

(8) $$\begin{array}{cc} Y_{B,j}= & \sum_{i=1}^{m_{3}}{\left(M_{m_{7}}\cdots M_{1}\right)}_{\zeta_{B}\left(j\right),i}X_{i}+\sum_{i^{\prime }=1}^{m_{5}}{\left(M_{m_{7}}^{\prime }\cdots M_{1}^{\prime }\right)}_{\zeta_{B}\left(j\right),\eta \left(i^{\prime }\right)}Z_{i^{\prime }} \end{array}$$

(9) $$\begin{array}{cc} Y_{E,j}= & \sum_{i=1}^{m_{3}}{\left(M_{m_{7}}\cdots M_{1}\right)}_{\zeta_{E}\left(j\right),i}X_{i}+\sum_{i^{\prime }=1}^{m_{5}}{\left(M_{m_{7}}^{\prime }\cdots M_{1}^{\prime }-I\right)}_{\zeta_{E}\left(j\right),\eta \left(i^{\prime }\right)}Z_{i^{\prime }}. \end{array}$$

When Eve eavesdrops the edges $E_{E}\cap E_{A}$, she obtains the information on $E_{E}\cap E_{A}$ before her noise injection. Hence, to express her obtained information on $E_{E}\cap E_{A}$, we need to subtract her injected information on $E_{E}\cap E_{A}$. Hence, we need −I in the second term of (9). We introduce the projection $P_{E,A}$ (an $m_{1}\times m_{5}$ matrix) as $P_{E,A;i,j}=\delta_{i,\eta (j)}$. Due to (8) and (9), the matrices $H_{B}:=P_{B}M_{m_{7}}^{\prime }\cdots M_{1}^{\prime }P_{E,A}$ and $H_{E}:=P_{E}\left(M_{m_{7}}^{\prime }\cdots M_{1}^{\prime }-I\right)P_{E,A}$ satisfy conditions (2) with the matrices $K_{B}$ and $K_{E}$, respectively. This model ($K_{B}$, $K_{E}$, $H_{B}$, $H_{E}$) to give (2) is called the wiretap and addition model determined by (V,E) and $\left(E_{E},E_{A},\theta \right)$, which expresses the topology and dynamics.

### 2.5. Strategy and Order of Communication

To discuss the active attack, we see how the causal condition for the subsets ${\left\{w_{i}\right\}}_{1,\ldots ,m_{5}}$ follows from the network topology in the wiretap and addition model. We choose the domain index subsets ${\left\{w_{i}\right\}}_{1\leq i\leq m_{5}}$ for α; i.e., Eve chooses the added error $Z_{i}$ on the edge $e\left(\eta \left(i\right)\right)\in E_{A}$ as a function $\alpha_{i}$ of the vector ${\left[Y_{E,j}\right]}_{j\in w_{i}}$. Since the order of Eve’s attack is characterized by the function η from $\left\{1,\ldots ,m_{5}\right\}$ to $E_{A}\subset \left\{1,\ldots ,m_{1}\right\}$, we discuss what condition for the pair $\left(\eta ,{\left\{w_{i}\right\}}_{i}\right)$ guarantees the causal condition for the subsets ${\left\{w_{i}\right\}}_{i}$.

First, one may assume that the tail node of the edge e(j) sends the information to the edge e(j) after the head node of the edge e(j−1) receives the information to the edge e(j−1). Since this condition determines the order of Eve’s attack, the function η must be a strictly increasing function from $\left\{1,\ldots ,m_{5}\right\}$ to $\left\{1,\ldots ,m_{1}\right\}$. Additionally, due to this time ordering, the subset $w_{i}$ needs to be $\left\{j|\eta \left(i\right)\geq \zeta_{E}\left(j\right)\right\}$ or its subset. We call these two conditions the full time-ordered condition for the function η and the subsets ${\left\{w_{i}\right\}}_{i}$. Since the function η is strictly increasing, condition (A2) for the causal condition holds. Since the relation (5) implies that $M_{m_{7}}^{\prime }\cdots M_{1}^{\prime }-I$ is a lower triangular matrix with zero diagonal elements, the strictly increasing property of η yields that

(10) $$\begin{array}{c} H_{E;j,i}=0\;\mathrm{when}\;\eta \left(i\right)\geq \zeta_{E}\left(j\right), \end{array}$$

which implies condition (A1) for the causal condition. In this way, the full time-ordered condition for the function η and the subsets ${\left\{w_{i}\right\}}_{i}$ satisfies the causal condition.

However, the full time ordered condition does not hold in general, even when we reorder the numbers assigned to the edges. That is, if the network is not well synchronized, Eve can make an attack across several channels; i.e., it is possible that Eve might intercept (i.e., wiretap and contaminate) the information of an edge before the head node of the previous edge receives the information on the edge. Hence, we consider the case when the partial time-ordered condition holds, but the full time-ordered condition does not necessarily hold. (For an example, we consider the following case. Eve gets the information on the first edge. Then, she gets the information on the second edge before she hands over the information on the first edge to the tail node of the first edge. In this case, she can change the information on the first edge based on the information on the first and second edges. Then, the time-ordered condition (10) does not hold.) That is, the function η from $\left\{1,\ldots ,m_{5}\right\}$ to *E* is injective but is not necessarily monotonically increasing. Given the matrix θ, we define the function $\gamma_{\theta }\left(j\right):=\min_{j^{\prime }}\left\{j^{\prime }|\theta_{j^{\prime },j}\neq 0\right\}$. Here, when no index $j^{\prime }$ satisfies the condition $\theta_{j^{\prime },j}\neq 0$, $\gamma_{\theta }\left(j\right)$ is defined to be $m_{1}+1$. Then, we say that the function η and the subsets ${\left\{w_{i}\right\}}_{i}$ are admissible under θ when {e(k)|k∈Imη}=EA, the subsets ${\left\{w_{i}\right\}}_{i}$ satisfy condition (A2) for the causal condition, and any element $j\in w_{i}$ satisfies

(11) $$\begin{array}{c} \zeta_{E}\left(j\right)<\gamma_{\theta }\left(\eta \left(i\right)\right). \end{array}$$

Here, Imη expresses the image of the function η. The condition (11) and the condition (5) imply the following condition; for $j\in w_{i}$, there is no sequence $\zeta_{E}\left(j\right)=j_{1}>j_{2},\ldots >j_{l}=\eta \left(i\right)$ such that

(12) $$\begin{array}{c} \theta_{j_{i},j_{i+1}}\neq 0. \end{array}$$

This condition implies condition (A1) for the causal condition. Since the admissibility under θ is natural, even when the full time-ordered condition does not hold, the causal condition can be naturally derived.

Given two admissible pairs $\left(\eta ,{\left\{w_{i}\right\}}_{i}\right)$ and $\left(\eta^{\prime },{\left\{w_{i}^{\prime }\right\}}_{i}\right)$, we say that the pair $\left(\eta ,{\left\{w_{i}\right\}}_{i}\right)$ is superior to $\left(\eta^{\prime },{\left\{w_{i}^{\prime }\right\}}_{i}\right)$ for Eve when $w_{{\eta^{\prime }}^{-1}\left(j\right)}^{\prime }\subset w_{\eta^{-1}\left(j\right)}$ for any $j\in E_{A}$. Now, we discuss the optimal choice of $\left(\eta ,{\left\{w_{i}\right\}}_{i}\right)$ in this sense when $E_{A}$ is given. That is, we choose the subset $w_{i}$ as large as possible under the admissibility under θ. Then, we choose the bijective function $\eta_{o}$ from $\left\{1,\ldots ,m_{5}\right\}$ to $E_{A}$ such that $\gamma_{\theta }\circ \eta_{o}$ is monotonically increasing. Then, we define $w_{o,i}:=\left\{j|\zeta_{E}\left(j\right)<\gamma_{\theta }\left(\eta_{o}\left(i\right)\right)\right\}$, which satisfies the admissibility under θ. conditions (A1) and (A2) for the causal condition. Further, when the pair $\left(\eta ,{\left\{w_{i}\right\}}_{i}\right)$ is admissible under θ, the condition (11) implies $w_{\eta^{-1}\left(j\right)}\subset w_{o,\eta_{o}^{-1}\left(j\right)}$ for $j\in E_{A}$; i.e., $w_{o,i}$ is the largest subset under the admissibility under θ. Hence, we obtain the optimality of $\left(\eta_{o},{\left\{w_{o,i}\right\}}_{i}\right)$. Although the choice of $\eta_{o}$ is not unique, the choice of $w_{o,\eta_{o}^{-1}\left(j\right)}$ for $j\in E_{A}$ is unique.

### 2.6. Secrecy in Concrete Network Model

**Figure 1:**
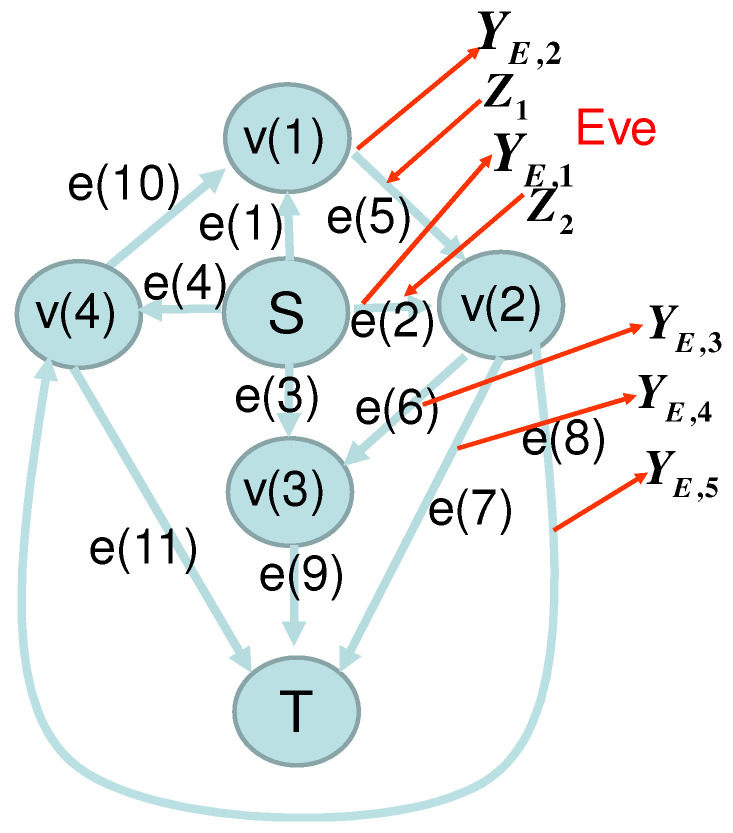
Network of Section 2.6 with name of edges.

**Figure 2:**
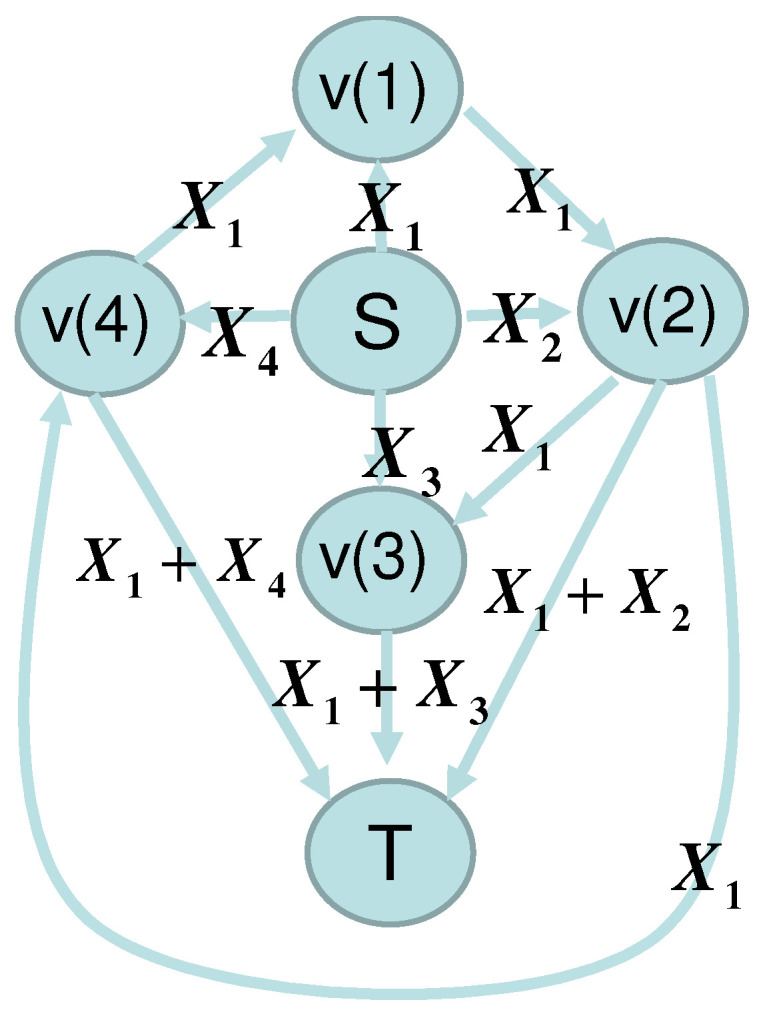
Network of Section 2.6 with network flow.

In this subsection, as an example, we consider the network given in Figure 1 and Figure 2, which shows that our framework can be applied to the network without synchronization. Alice sends the variables $X_{1},\ldots ,X_{4}\in F_{q}$ to nodes v(1),v(2),v(3), and v(4) via the edges e(1),e(2),e(3), and e(4), respectively. The edges e(5),e(6),e(8),e(10) send the elements received from the edges e(1),e(5),e(5),e(8), respectively. The edges e(7), e(9), and e(11) send the sum of two elements received from the edge pairs (e(2),e(5)),(e(3),e(6)), and (e(4),e(8)), respectively.

Bob receives elements via the edges e(7),e(9), and e(11), which are written as $Y_{B,1},Y_{B,2},$ and $Y_{B,3}$, respectively. Then, the matrix $K_{B}$ is given as

(13) $$\begin{array}{c} K_{B}=\left(\begin{array}{cccc} 1 & 1 & 0 & 0\\ 1 & 0 & 1 & 0\\ 1 & 0 & 0 & 1 \end{array}\right). \end{array}$$

Then, $m_{3}=4$ and $m_{4}=3$.

Now, we assume that Eve eavesdrops the edges e(2),e(5),e(6),e(7), and e(8), i.e., all edges connected to v(2), and contaminates the edge e(2),e(5). Then, we set $\zeta_{B}\left(1\right)=7,\zeta_{B}\left(2\right)=9,\zeta_{B}\left(3\right)=11$ and $\zeta_{E}\left(1\right)=2,\zeta_{E}\left(2\right)=5,\zeta_{E}\left(3\right)=6,\zeta_{E}\left(4\right)=7,\zeta_{E}\left(5\right)=8$. Eve can choose the function η as

(14) $$\begin{array}{c} \eta (1)=5,\;\eta (2)=2 \end{array}$$

while η(1)=2,η(2)=5 is possible. In the following, we choose (14). Since $\gamma_{\theta }\left(2\right)=7$ and $\gamma_{\theta }\left(5\right)=6$, the subsets $w_{i}$ are given as

(15) $$\begin{array}{c} w_{1}:=w_{o,1}=\left\{1,2\right\},\;w_{2}:=w_{o,2}=\left\{1,2,3\right\} \end{array}$$

This case satisfies conditions (A1) and (A2). Hence, this model satisfies the causal condition. Lemma 1 guarantees that any strategy also satisfies the uniqueness condition.

We denote the observed information on the edges e(2),e(5),e(6),e(7), and e(8) by $Y_{E,1},Y_{E,2},Y_{E,3},Y_{E,4},$ and $Y_{E,5}$. As in Figure 1, Eve adds $Z_{1}$ and $Z_{2}$ in edges e(2) and e(5). Then, the matrices $H_{B}$, $K_{E}$, and $H_{E}$ are given as

(16) $$\begin{array}{cc} H_{B} & =\left(\begin{array}{cc} 1 & 1\\ 0 & 0\\ 0 & 0 \end{array}\right),\;K_{E}=\left(\begin{array}{cccc} 0 & 1 & 0 & 0\\ 1 & 0 & 0 & 0\\ 1 & 0 & 0 & 0\\ 1 & 1 & 0 & 0\\ 1 & 0 & 0 & 0 \end{array}\right),\;H_{E}=\left(\begin{array}{cc} 0 & 0\\ 0 & 0\\ 0 & 1\\ 1 & 1\\ 0 & 1 \end{array}\right). \end{array}$$

In this case, to keep the secrecy of the message to be transmitted, Alice and Bob can use coding as follows. When Alice’s message is $M\in F_{q}$, Alice prepares scramble random number $L_{1},L_{2},L_{3}\in F_{q}$. These variables are assumed to be subject to the uniform distribution independently. She encodes them as $X_{i}=L_{i}$ for i=1,…,3 and $X_{4}=-M+L_{1}+L_{2}+L_{3}$. As shown in the following, under this code, Eve cannot obtain any information for *M*, even though she makes active attack. Due to Theorem 1, it is sufficient to show the secrecy when $Z_{i}=0$. Since Eve’s information is $Y_{E,1}=X_{2},Y_{E,2}=X_{1},Y_{E,3}=X_{1},Y_{E,4}=X_{1}+X_{2}$, and $Y_{E,5}=X_{1}$, the matrix *A* given in Section 2.2 is

(17) $$\begin{array}{c} \left(\begin{array}{cccc} 0 & 0 & 1 & 0\\ 0 & 1 & 0 & 0\\ 0 & 1 & 0 & 0\\ 0 & 1 & 1 & 0\\ 0 & 1 & 0 & 0 \end{array}\right). \end{array}$$

Thus, Proposition 2 guarantees that Eve cannot obtain any information for the message *M*.

Indeed, the above attack can be considered as the following. Eve can eavesdrop all edges connected to the intermediate node v(2) and contaminate all edges incoming to the intermediate node v(2). Hence, it is natural to assume that Eve similarly eavesdrops and contaminates at another intermediate node v(i). That is, Eve can eavesdrop all edges connected to the intermediate node v(i) and contaminate all edges incoming to the intermediate node v(i). For all nodes v(i), this code has the same secrecy against the above Eve’s attack for node v(i).

Furthermore, the above code has the secrecy even when the following attack.

**(B1)**  Eve eavesdrops one of three edges e(7),e(9), and e(11) connected to the sink node, and eavesdrops and contaminates one of the remaining eight edges e(1),e(2),e(3),e(4),e(5),e(6),e(8), and e(10) that are not connected to the sink node.

To apply Corollary 1 for analysis of the secrecy, we denote the low vector in Corollary 1 corresponding to the edge e(i) by $v_{i}$. Then, the vectors $v_{7}$, $v_{9}$, and $v_{11}$ are (0,1,1,0), (0,1,0,1), and (−1,2,1,1). The remaining vectors $v_{1}$, $v_{2}$, $v_{3}$, $v_{4}$, $v_{5}$, $v_{6}$, $v_{8}$, and $v_{10}$ are (0,1,0,0), (0,0,1,0), (0,0,0,1), (−1,1,1,1), (0,1,0,0), (0,1,0,0), (0,1,0,0), and (0,1,0,0). Since any combination of the vector of the first group and the second group cannot be (1,0,0,0) the combination of Corollary 1 and Theorem 2 guarantees that the secrecy holds under the above attack (B1).

### 2.7. A Problem in Error Detection in a Concrete Network Model

However, the network given in Figure 1 and Figure 2 has the problem for the detection of the error in the following meaning. When Eve makes an active attack, Bob’s recovering message is different from the original message due to the contamination. Further, Bob cannot detect the existence of the error in this case. It is natural to require the detection of the existence of the error when the original message cannot be recovered and the secrecy. As a special attack model, we consider the following scenario with the attack (B1).

**(B2)**  Our node operations are fixed to the way as Figure 2.
**(B3)**  The message set M and all information on all edges are $F_{2}$.
**(B4)**  The variables $X_{1},X_{2},X_{3},$ and $X_{4}$ are given as the output of the encoder. The encoder on the source node can be chosen, but is restricted to linear. It is allowed to use a scramble random number, which is an element of $L:=F_{2}^{k}$ with a certain integer *k*. Formally, the encoder is given as a linear function from M×L to $F_{2}^{4}$.
**(B5)**  The decoder on the sink node can be chosen dependently of the encoder and independently of Eve’s attack.

Then, it is impossible to make a pair of an encoder and a decoder such that the secrecy holds and Bob can detect the existence of error.

This fact can be shown as follows. In order to detect it, as discussed in Section 2.2, Alice needs to make an encoder such that the vector $\left(Y_{B,1},Y_{B,2},Y_{B,3}\right)$ belongs to a linear subspace because the detection can be done only by observing that the vector does not belong to a certain linear subspace, which can be written as $\left\{\left(Y_{B,1},Y_{B,2},Y_{B,3}\right)|c_{1}Y_{B,1}+c_{2}Y_{B,2}+c_{3}Y_{B,3}=0\right\}$ with a non-zero vector $\left(c_{1},c_{2},c_{3}\right)\in F_{2}^{3}$. That is, the encoder needs to be constructed so that the relation $c_{1}Y_{B,1}+c_{2}Y_{B,2}+c_{3}Y_{B,3}=\left(c_{1}+c_{2}+c_{3}\right)X_{1}+c_{1}X_{2}+c_{2}X_{3}+c_{3}X_{4}=0$ holds unless Eve’s injection is made. Since our field is $F_{2}^{3}$, we have three cases. (C1) $\left(c_{1},c_{2},c_{3}\right)$ is (1,0,0), (0,1,0), or (0,0,1). (C2) $\left(c_{1},c_{2},c_{3}\right)$ is (1,1,0), (0,1,1), or (0,1,1). (C3) $\left(c_{1},c_{2},c_{3}\right)$ is (1,1,1). If we impose another linear condition, the transmitted information is restricted into a one-dimensional subspace, which means that the message *M* uniquely decides the vector $\left(Y_{B,1},Y_{B,2},Y_{B,3}\right)$. Hence, if Eve eavesdrops one suitable variable among three variables $Y_{B,1},Y_{B,2},$ and $Y_{B,3}$, Eve can infer the original message.

In the first case (C1), one of three variables $Y_{B,1},Y_{B,2},$ and $Y_{B,3}$ is zero unless Eve’s injection is made. When $Y_{B,1}=0$, i.e., $\left(c_{1},c_{2},c_{3}\right)=\left(1,0,0\right)$, Bob can detect an error on the edge e(5) or e(2) because the error on e(5) or e(2) affects $Y_{B,1}$ so that $Y_{B,1}$ is not zero. However, Bob cannot detect any error on the edge e(4) because the error does not affect $Y_{B,1}$. The same fact can be applied to the case when $Y_{B,2}=0$. When $Y_{B,3}=0$, Bob cannot detect any error on the edge e(3) because the error does not affect $Y_{B,3}$.

In the second case (C2), two of three variables $Y_{B,1},Y_{B,2},$ and $Y_{B,3}$ have the same value unless Eve’s injection is made. When $Y_{B,1}=Y_{B,2}$, i.e., $\left(c_{1},c_{2},c_{3}\right)=\left(1,1,0\right)$, Bob can detect an error on the edge e(2) or e(3) because the error on e(2) or e(3) affects $Y_{B,1}$ or $Y_{B,2}$ so that $Y_{B,1}+Y_{B,2}$ is not zero. However, Bob cannot detect any error on the edge e(4) because the error does not affect $Y_{B,1}$ or $Y_{B,2}$. Similarly, When $Y_{B,2}=Y_{B,3}$ ($Y_{B,1}=Y_{B,3}$), Bob cannot detect any error on the edge e(2) (e(3)).

In the third case (C3), the relation $Y_{B,1}=Y_{B,2}+Y_{B,3}$ holds; i.e., $\left(c_{1},c_{2},c_{3}\right)=\left(1,1,1\right)$. Then, the linearity of the code implies that the message has the form $a_{1}Y_{B,1}+a_{2}Y_{B,2}+a_{3}Y_{B,3}$. Due to the relation $Y_{B,1}=Y_{B,2}+Y_{B,3}$, the value $a_{1}Y_{B,1}+a_{2}Y_{B,2}+a_{3}Y_{B,3}=\left(a_{1}+a_{2}\right)Y_{B,2}+\left(a_{1}+a_{3}\right)Y_{B,3}$ is limited to $Y_{B,1}$, $Y_{B,2}$, $Y_{B,3}$, or 0 because our field is $F_{2}$. Since the message is not a constant, it is limited to one of $Y_{B,1}$, $Y_{B,2}$, or $Y_{B,3}$. Hence, when it is $Y_{B,1}$, Eve can obtain the message by eavesdropping the edge e(7). In other cases, Eve can obtain the message in the same way.

To resolve this problem, we need to use this network multiple times. Hence, in the next section, we discuss the case with multiple transmission.

### 2.8. Wiretap and Replacement Model

In the above subsections, we have discussed the case when Eve injects the noise in the edges $E_{A}$ and eavesdrops the edges $E_{E}$. In this subsection, we assume that $E_{A}\subset E_{E}$ and Eve eavesdrops the edges $E_{E}$ and replaces the information on the edges $E_{A}$ by other information. While this assumption implies $m_{5}\leq m_{6}$ and the image of η is included in the image of $\zeta_{E}$, the function η does not necessarily equal the function $\zeta_{E}$ because the order that Eve sends her replaced information to the heads of edges does not necessarily equal the order that Eve intercepts the information on the edges. Additionally, this case belongs to general wiretap and addition model (2) as follows. Modifying the matrix $M_{j}$, we define the new matrix $M_{j}^{\prime \prime }$ as follows. When there is an index *i* such that $\zeta_{E}\left(i\right)=j$, the $j+m_{3}$-th row vector of the new matrix $M_{j}^{\prime \prime }$ is defined by ${\left[\delta_{j+m_{3},j^{\prime }}\right]}_{1\leq j^{\prime }\leq m_{1}}$ and the remaining part of $M_{j}^{\prime \prime }$ is defined as the identity matrix. Otherwise, $M_{j}^{\prime \prime }$ is defined to be $M_{j}$. Additionally, we define another matrix *F* as follows. The $\zeta_{E}\left(i\right)$-th row vector of the new matrix *F* is defined by ${\left[\theta_{\zeta_{E}\left(i\right),j^{\prime }}\right]}_{1\leq j^{\prime }\leq m_{1}}$ and the remaining part of *F* is defined as the identity matrix. Hence, we have

(18) $$\begin{array}{cc} Y_{B,j}= & \sum_{i=1}^{m_{3}}{\left(M_{m_{7}}^{\prime \prime }\cdots M_{1}^{\prime \prime }\right)}_{\zeta_{B}\left(j\right),i}X_{i}+\sum_{i^{\prime }=1}^{m_{5}}{\left(M_{m_{7}}^{\prime \prime }\cdots M_{1}^{\prime \prime }\right)}_{\zeta_{B}\left(j\right),\eta \left(i^{\prime }\right)}Z_{i^{\prime }} \end{array}$$

(19) $$\begin{array}{cc} Y_{E,j}= & \sum_{i=1}^{m_{3}}{\left(FM_{m_{7}}^{\prime \prime }\cdots M_{1}^{\prime \prime }\right)}_{\zeta_{E}\left(j\right),i}X_{i}+\sum_{i^{\prime }=1}^{m_{5}}{\left(FM_{m_{7}}^{\prime \prime }\cdots M_{1}^{\prime \prime }\right)}_{\zeta_{E}\left(j\right),\eta \left(i^{\prime }\right)}Z_{i^{\prime }}. \end{array}$$

Then, we choose matrices $K_{B}^{\prime }$, $K_{E}^{\prime }$, $H_{B}^{\prime }$, and $H_{E}^{\prime }$ as $K_{B}^{\prime }:=P_{B}M_{m_{7}}^{\prime \prime }\cdots M_{1}^{\prime \prime }P_{A}$, $K_{E}^{\prime }:=P_{E}FM_{m_{7}}^{\prime \prime }\cdots M_{1}^{\prime \prime }P_{A}$, $H_{B}^{\prime }:=P_{B}M_{m_{7}}^{\prime \prime }\cdots M_{1}^{\prime \prime }P_{E}^{T}$, and $H_{E}^{\prime }:=P_{E}FM_{m_{7}}^{\prime \prime }\cdots M_{1}^{\prime \prime }P_{E}^{T}$, which satisfy conditions (2) due to (18) and (19). This model ($K_{B}^{\prime }$, $K_{E}^{\prime }$, $H_{B}^{\prime }$, $H_{E}^{\prime }$) is called the wiretap and replacement model determined by (V,E) and $\left(E_{E},E_{A},\theta ,\eta \right)$. Notice that the projections $P_{A},P_{B},$ and $P_{E}$ are defined in Section 2.3.

Next, we discuss the strategy $\alpha^{\prime }$ under the matrices $K_{B}^{\prime }$, $K_{E}^{\prime }$, $H_{B}^{\prime }$, and $H_{E}^{\prime }$ such that the added error $Z_{i}$ is given as a function $\alpha_{i}^{\prime }$ of the vector ${\left[Y_{E,j}\right]}_{j\in w_{i}}$. Since the decision of the injected noise does not depend on the results of the decision, we impose the causal condition defined in Definition 4 for the subsets $w_{i}$.

When the relation $j\in w_{i}$ holds with $\zeta_{E}\left(j\right)=\eta \left(i\right)$, a strategy $\alpha^{\prime }$ on the wiretap and replacement model ($K_{B}^{\prime }$, $K_{E}^{\prime }$, $H_{B}^{\prime }$, $H_{E}^{\prime }$) determined by (V,E) and $\left(E_{E},\theta \right)$ is written by another strategy α on the wiretap and addition model $K_{B}$, $K_{E}$, $H_{B}$, and $H_{E}$ determined by (V,E) and $\left(E_{E},\theta \right)$, which is defined as $\alpha_{j}\left({\left[Y_{E,j^{\prime }}\right]}_{j^{\prime }\in w_{i}}\right):=\alpha_{j}^{\prime }\left({\left[{\hat{Y}}_{E,j^{\prime }}\right]}_{j^{\prime }\in w_{i}}\right)-Y_{E,j}$. In particular, due to the condition (5), the optimal choice $\eta_{o},\left\{w_{o,i}\right\}$ under the partial time-ordered condition satisfies that the relation $j\in w_{o,i}$ holds with $\zeta_{E}\left(j\right)=\eta_{o}\left(i\right)$. That is, under the partial time-ordered condition, the strategy on the wiretap and replacement model can be written by another strategy on the wiretap and addition model.

However, if there is no synchronization among vertexes, Eve can inject the replaced information to the head of an edge before the tail of the edge sends the information to the edge. Then, the partial time-ordered condition does not hold. In this case, the relation $j\in w_{i}$ does not necessarily hold with $\zeta_{E}\left(j\right)=\eta \left(i\right)$. Hence, a strategy $\alpha^{\prime }$ on the wiretap and replacement model ($K_{B}^{\prime }$, $K_{E}^{\prime }$, $H_{B}^{\prime }$, $H_{E}^{\prime }$) cannot be necessarily written as another strategy on the wiretap and addition model ($K_{B}$, $K_{E}$, $H_{B}$, $H_{E}$).

**Figure 3:**
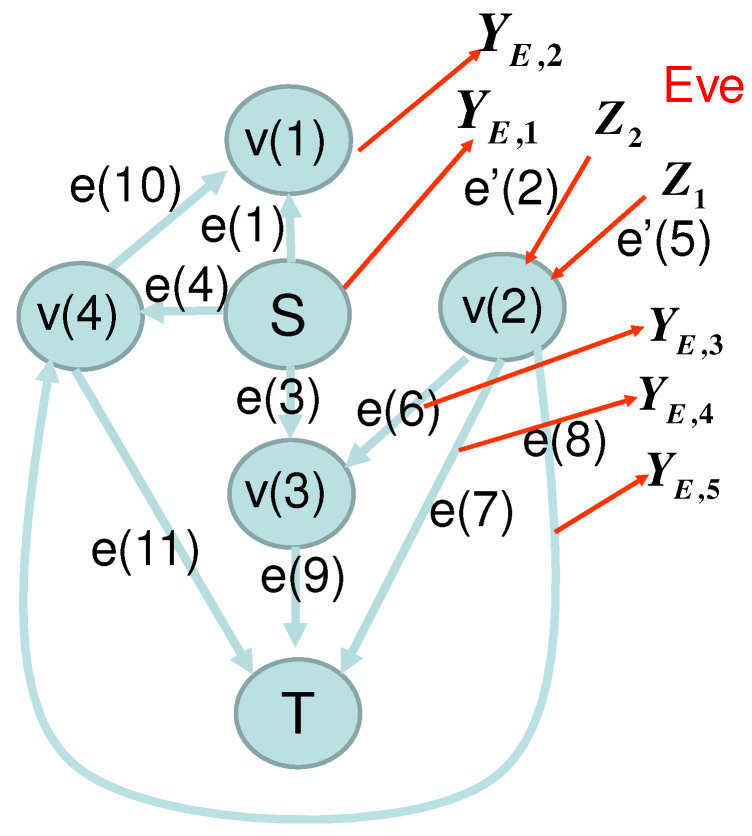
Network of Section 2.6 with the wiretap and replacement model. Eve injects the replaced information on the edges $e^{\prime }\left(2\right)$ and $e^{\prime }\left(5\right)$.

To see this fact, we discuss an example given in Section 2.6. In this example, the network structure of the wiretap and replacement model is given by Figure 3.

## 3. Multiple Transmission Setting

### 3.1. General Model

Now, we consider the *n*-transmission setting, where Alice uses the same network *n* times to send a message to Bob. Alice’s input variable (Eve’s added variable) is given as a matrix $X^{n}=\left(X_{1},\ldots ,X_{n}\right)\in F_{q}^{m_{3}\times n}$ (a matrix $Z^{n}=\left(Z_{1},\ldots ,Z_{n}\right)\in F_{q}^{m_{5}\times n}$), and Bob’s (Eve’s) received variable is given as a matrix $Y_{B}^{n}=\left(Y_{B,1},\ldots ,Y_{B,n}\right)\in F_{q}^{m_{4}\times n}$ (a matrix $Y_{E}^{n}=\left(Y_{E,1},\ldots ,Y_{E,n}\right)\in F_{q}^{m_{6}\times n}$). Then, we consider the model

(20) $$\begin{array}{cc} Y_{B}^{n} & =K_{B}X^{n}+H_{B}Z^{n}, \end{array}$$

(21) $$\begin{array}{cc} Y_{E}^{n} & =K_{E}X^{n}+H_{E}Z^{n}, \end{array}$$

whose realization in a concrete network will be discussed in Section 3.2 and Section 3.3. Notice that the relations (20) and (21) with $H_{E}=0$ (only the relation (20)) were treated as the starting point of the paper [20] (the papers [22,23,24]).

In this case, regarding *n* transmissions of one channel as *n* different edges, we consider the directed graph composed of $nm_{5}$ edges. Then, Eve’s strategy $\alpha^{n}$ is given as $nm_{5}$ functions ${\left\{\alpha_{i,l}\right\}}_{1\leq i\leq m_{5},1\leq l\leq n}$ from $Y_{E}^{n}$ to the respective components of $Z^{n}$. In this case, we extend the uniqueness condition to the *n*-transmission version.

**Definition** **3.** *For any value of $K_{E}x^{n}$, there uniquely exists $y^{n}\in F_{q}^{m_{6}\times n}$ such that*

(22) $$\begin{array}{c} y^{n}=K_{E}x^{n}+H_{E}\alpha^{n}\left(y^{n}\right). \end{array}$$

*This condition is called the n-uniqueness condition.*

Since we have *n* transmissions on each channel, the matrix θ is given as an $\left(nm_{1}\right)\times \left(nm_{1}\right)$ matrix. In the following, we see how the matrix θ is given and how the *n*-uniqueness condition is satisfied in a more concrete setting.

### 3.2. The Multiple Transmission Setting with Sequential Transmission

This section discusses how the model given in Section 3.1 can be realized in the case with sequential transmission as follows. Alice sends the first information $X_{1}$. Then, Alice sends the second information $X_{2}$. Alice sequentially sends the information $X_{3},\ldots ,X_{n}$. Hence, when an injective function $\tau_{E}$ from $\left\{1,\ldots ,m_{1}\right\}\times \left\{1,\ldots ,n\right\}$ to $\left\{1,\ldots ,nm_{1}\right\}$ gives the time ordering of $nm_{1}$ edges, it satisfies the condition

(23) $$\begin{array}{c} \tau_{E}\left(i,l\right)\leq \tau_{E}\left(i^{\prime },l^{\prime }\right)\;\mathrm{when}\;i\leq i^{\prime }\wedge l\leq l^{\prime }. \end{array}$$

Here, we assume that the topology and dynamics of the network and the edge attacked by Eve do not change during *n* transmissions, which is called the stationary condition. All operations in intermediate nodes are linear. Additionally, we assume that the time ordering on the network flow does not cause any correlation with the delayed information like Figure 1 unless Eve’s injection is made; i.e., the *l*-th information $Y_{B,l}$ received by Bob is independent of $X_{1},\ldots ,X_{l-1},X_{l+1},\ldots ,X_{n}$, which is called the independence condition. The independence condition means that there is no correlation with the delayed information. Due to the stationary and independence conditions, the $\left(nm_{1}\right)\times \left(nm_{1}\right)$ matrix θ satisfies that

(24) $$\begin{array}{c} \theta_{\left(i,l),(j,k\right)}={\bar{\theta }}_{i,j}\delta_{k,l}, \end{array}$$

where ${\bar{\theta }}_{i,j}:=\theta_{\left(i,1),(j,1\right)}$. When the $m_{1}\times m_{1}$ matrix $\bar{\theta }$ satisfies the partial time-ordered condition (5), due to (23) and (24), the $\left(nm_{1}\right)\times \left(nm_{1}\right)$ matrix θ satisfies the partial time-ordered condition (5) with respect to the time ordering $\tau_{E}$. Since the stationary condition guarantees that the edges attacked by Eve do not change during *n* transmissions, the above condition for θ implies the model (20) and (21). This scenario is called the *n*-sequential transmission.

Since the independence condition is not so trivial, it is needed to discuss when it is satisfied. If the *l*-th transmission has no correlation with the delayed information of the previous transmissions for l=2,…,n, the independence condition holds. In order to satisfy the above independence condition, the acyclic condition for the network graph is often imposed. This is because any causal time ordering on the network flow does not cause any correlation with the delayed information and achieves the max-flow if the network graph has no cycle [31]. In other words, if the network graph has a cycle, there is a possibility that a good time ordering on the network flow that causes correlation with the delayed information. However, there is no relation between the relations (20) and (21) and the acyclic condition for the network graph, and the relations (20) and (21) directly depend on the time ordering on the network flow. That is, the acyclic condition for the network graph is not equivalent to the existence of the effect of delayed information. Indeed, if we employ breaking cycles on intermediate nodes ([31] Example 3.1), even when the network graph has cycles, we can avoid any correlation with the delayed information. (To handle a time ordering with delayed information, one often employs a convolution code [32]. It is used in sequential transmission, and requires synchronization among all nodes. Additionally, all the intermediate nodes are required to make a cooperative coding operation under the control of the sender and the receiver. If we employ breaking cycles, we do not need such synchronization and avoid any correlation with the delayed information.) Additionally, see the example given in Section 3.5.

To extend the causality condition, we focus on the domain index subsets ${\left\{w_{i,l}\right\}}_{1\leq i\leq m_{5},1\leq l\leq n}$ of $\left\{1,\ldots ,m_{6}\right\}\times \left\{1,\ldots ,n\right\}$ for Eve’s strategy $\alpha^{n}={\left\{\alpha_{i,l}\right\}}_{1\leq i\leq m_{5},1\leq l\leq n}$. Then, we define the causality condition under the order function $\tau_{E}$.

**Definition** **4.** *We say that the domain index subsets ${\left\{w_{i,l}\right\}}_{i,l}$ satisfy the n-causal condition under the order function $\tau_{E}$ and the function η from $\left\{1,\ldots ,m_{5}\right\}$ to $\left\{1,\ldots ,m_{1}\right\}$ when the following two conditions hold:*

**(A1’)**  *The relation $H_{E;j,i}=0$ holds for $\left(j,l\right)\notin w_{i,l}$.*
**(A2’)**  *The relation $w_{i,l}\subseteq w_{i^{\prime },l^{\prime }}$ holds when $\tau_{E}\left(\eta \left(i\right),l\right)\leq \tau_{E}\left(\eta \left(i^{\prime }\right),l^{\prime }\right)$.*

Next, we focus on the domain index subsets ${\left\{w_{i,l}\right\}}_{i,l}$ and the function η from $\left\{1,\ldots ,m_{5}\right\}$ to $\left\{1,\ldots ,m_{1}\right\}$. We say that the pair $\left(\eta ,{\left\{w_{i,l}\right\}}_{i,l}\right)$ are *n*-admissible under $\bar{\theta }$ under the order function $\tau_{E}$ when {e(k)|k∈Imη}=EA, the subsets ${\left\{w_{i,l}\right\}}_{i,l}$ satisfy condition (A2’) for the *n* causal condition, and any element $\left(j,l^{\prime }\right)\in w_{i,l}$ satisfies

(25) $$\begin{array}{c} \tau_{E}\left(\zeta_{E}\left(j\right),l^{\prime }\right)<\gamma_{\bar{\theta }}\left(\eta \left(i\right),l\right). \end{array}$$

where the function $\gamma_{\bar{\theta }}$ is defined as

(26) $$\begin{array}{c} \gamma_{\bar{\theta }}\left(j,l\right):=\min_{j^{\prime }}\left\{\tau_{E}\left(j^{\prime },l\right)|{\bar{\theta }}_{j^{\prime },j}\neq 0\right\}. \end{array}$$

Here, when no index $j^{\prime }$ satisfies the condition ${\bar{\theta }}_{j^{\prime },j}\neq 0$, $\gamma_{\bar{\theta }}\left(j,l\right)$ is defined to be $nm_{1}+1$. In the same way as Section 2.5, we find that the *n*-admissibility of the pair $\left(\eta ,{\left\{w_{i,l}\right\}}_{i,l}\right)$ implies the *n*-causal condition under $\tau_{E}$ and η for the domain index subsets ${\left\{w_{i,l}\right\}}_{i,l}$.

Given two *n*-admissible pairs $\left(\eta ,{\left\{w_{i,l}\right\}}_{i,l}\right)$ and $\left(\eta^{\prime },{\left\{w_{i,l}^{\prime }\right\}}_{i,l}\right)$, we say that the pair $\left(\eta ,{\left\{w_{i,l}\right\}}_{i,l}\right)$ is superior to $\left(\eta^{\prime },{\left\{w_{i,l}^{\prime }\right\}}_{i,l}\right)$ for Eve when $w_{{\eta^{\prime }}^{-1}\left(j\right),l}^{\prime }\subset w_{\eta^{-1}\left(j\right),l}$ for $j\in E_{A}$ and l=1,…,n. Then, we choose the bijective function $\tau_{E,\eta }$ from $\left\{1,\ldots ,m_{5}\right\}\times \left\{1,\ldots ,n\right\}$ to $\left\{1,\ldots ,nm_{5}\right\}$ such that $\gamma_{\bar{\theta }}\circ \eta \circ \tau_{E,\eta }^{-1}$ is monotonically increasing, where $\gamma_{\bar{\theta }}\circ \eta$ is defined as $\gamma_{\bar{\theta }}\circ \eta \left(i,l\right)=\gamma_{\bar{\theta }}\left(\eta \left(i\right),l\right)$. The function $\tau_{E,\eta }$ expresses the order of Eve’s contamination. Then, we define $w_{\eta ,i,l}:=\left\{\left(j,l^{\prime }\right)|\tau_{E}\left(\zeta_{E}\left(j\right),l^{\prime }\right)<\gamma_{\bar{\theta }}\left(\eta \left(i\right),l\right)\right\}$, which satisfies the *n*-admissibility under $\bar{\theta }$ and the order function $\tau_{E}$.

Further, when the pair $\left(\eta^{\prime },{\left\{w_{i,l}\right\}}_{i,l}\right)$ is *n*-admissible under $\bar{\theta }$ and $\tau_{E}$, the condition (25) implies $w_{{\eta^{\prime }}^{-1}\left(j\right),l}\subset w_{\eta ,\eta^{-1}\left(j\right),l}$ for $j\in E_{A}$ and l=1,…,n; i.e., $w_{\eta ,i,l}$ is the largest subset under the *n* admissibility under $\bar{\theta }$ and $\tau_{E}$. Hence, we obtain the optimality of $\left(\eta ,{\left\{w_{\eta ,i,l}\right\}}_{i,l}\right)$ when $\bar{\theta }$, $\tau_{E}$, and $E_{A}$ are given. Although the choice of η is not unique, the choice of $w_{\eta ,\eta^{-1}\left(j\right),l}$ for $j\in E_{A}$ and l=1,…,n is unique when $\bar{\theta }$, $\tau_{E}$, and $E_{A}$ are given.

In the same way as Lemma 1, we find that the *n*-causal condition with sequential transmission guarantees the *n*-uniqueness condition as follows.

**Lemma** **2.** *When a strategy α for the n-sequential transmission has domain index subsets to satisfy the n-causal condition, the strategy α satisfies the n-uniqueness condition.*

**Proof.**  Consider a big graph composed of $nm_{1}$ edges ${\left\{e\left(i,l\right)\right\}}_{1\leq i\leq m_{1},1\leq l\leq n}$ and $nm_{2}$ vertecies ${\left\{v\left(j,l\right)\right\}}_{1\leq j\leq m_{2},1\leq l\leq n}$. In this big graph, the coefficient matrix is given in (24). We assign the $nm_{1}$ edges the number $\tau_{E}\left(i,l\right)$. The *n*-causal and *n*-uniqueness conditions correspond to the causal and uniqueness conditions of this big network, respectively. Hence, Lemma 1 implies Lemma 2. □

### 3.3. Multiple Transmission Setting with Simultaneous Transmission

We consider anther scenario to realize the model given in Section 3.1. Usually, we employ an error correcting code for the information transmission on the edges in our graph. For example, when the information transmission is done by wireless communication, an error correcting code is always applied. Now, we assume that the same error correcting code is used on all the edges. Then, we set the length *n* to be the same value as the transmitted information length of the error correcting code. In this case, *n* transmissions are done simultaneously in each edge. Each node makes the same node operation for *n* transmissions, which implies the condition (24) for the $\left(nm_{1}\right)\times \left(nm_{1}\right)$ matrix θ. Then, the relations (20) and (21) hold because the delayed information does not appear. This scenario is called the *n*-simultaneous transmission.

In fact, when we focus on the mathematical aspect, the *n*-simultaneous transmission can be regarded as a special case of the *n*-sequential transmission. In this case, the independence condition always holds even when the network has a cycle. Further, the *n*-uniqueness condition can be derived in a simpler way without discussing the *n*-causal condition as follows.

In this scenario, given a function η from $\left\{1,\ldots ,m_{5}\right\}$ to $E_{A}\subset \left\{1,\ldots ,m_{1}\right\}$, Eve chooses the added errors $\left(Z_{i,1},\ldots ,Z_{i,n}\right)\in F_{q}^{n}$ on the edge $e\left(\eta \left(i\right)\right)\in E_{A}$ as a function $\alpha_{i}$ of the vector ${\left[Y_{E,j}\right]}_{j\in w_{i}}$ with subsets ${\left\{w_{i}\right\}}_{1\leq i\leq m_{5}}$ of $\left\{1,\ldots ,m_{6}\right\}$. Hence, in the same way as the single transmission, domain index subsets for α are given as subsets $w_{i}\subset \left\{1,\ldots ,m_{6}\right\}$ for $i\in \{1,\ldots ,m_{5}\}$. In the same way as Lemma 1, we have the following lemma.

**Lemma** **3.** *When a strategy α for the n-simultaneous transmission has domain index subsets to satisfy the causal condition, the strategy α satisfies the n-uniqueness condition.*

In addition, the wiretap and replacement model in this setting can be introduced for the *n*-sequential transmission and the *n*-simultaneous transmission in the same way as Section 2.8.

### 3.4. Non-Local Code and Reduction Theorem

Now, we assume only the model (20) and (21) and the *n*-uniqueness condition. Since the model (20) and (21) is given, we manage only the encoder in the sender and the decoder in the receiver. Although the operations in the intermediate nodes are linear and operate only on a single transmission, the encoder and the decoder operate across several transmissions. Such a code is called a non-local code to distinguish operations over a single transmission. Here, we formulate a non-local code to discuss the secrecy. Let M and L be the message set and the set of values of the scramble random number, which is often called the private randomness. Then, an encoder is given as a function $\phi_{n}$ from M×L to $F_{q}^{m_{3}\times n}$, and the decoder is given as $\psi_{n}$ from $F_{q}^{m_{4}\times n}$ to M. Here, the linearity for $\phi_{n}$ and $\psi_{n}$ is not assumed. That is, the decoder does not use the scramble random number *L* because it is not shared with the decoder. Our non-local code is the pair $\left(\phi_{n},\psi_{n}\right)$, and is denoted by $\Phi_{n}$. Then, we denote the message and the scramble random number as *M* and *L*. The cardinality of M is called the size of the code and is denoted by $|\Phi_{n}|$. More generally, when we focus on a sequence $\left\{l_{n}\right\}$ instead of {n}, an encoder $\phi_{n}$ is a function from M×L to $F_{q}^{m_{3}\times l_{n}}$, and the decoder $\psi_{n}$ is a function from $F_{q}^{m_{4}\times l_{n}}$ to M.

Here, we treat $K_{B},K_{E},H_{B}$, and $H_{E}$ as deterministic values, and denote the pairs $\left(K_{B},K_{E}\right)$ and $\left(H_{B},H_{E}\right)$ by K and H, respectively, while Alice and Bob might not have the full information for $K_{E},H_{B},$ and $H_{E}$. Additionally, we assume that the matrices K and H are not changed during transmission. In the following, we fix $\Phi_{n},K,H,\alpha^{n}$. As a measure of the leaked information, we adopt the mutual information $I(M;Y_{E}^{n},Z^{n})$ between *M* and Eve’s information $Y_{E}^{n}$ and $Z^{n}$. Since the variable $Z^{n}$ is given as a function of $Y_{E}^{n}$, we have $I\left(M;Y_{E}^{n},Z^{n}\right)=I\left(M;Y_{E}^{n}\right)$. Since the leaked information is given as a function of $\Phi_{n},K,H,\alpha^{n}$ in this situation, we denote it by $I\left(M;Y_{E}^{n}\right)\left[\Phi_{n},K,H,\alpha^{n}\right]$.

**Definition** **5.** *When we always choose $Z^{n}=0$, the attack is the same as the passive attack. This strategy is denoted by $\alpha^{n}=0$.*

When K,H are treated as random variables independent of M,L, the leaked information is given as the expectation of $I\left(M;Y_{E}^{n}\right)\left[\Phi_{n},K,H,\alpha^{n}\right]$. This probabilistic setting expresses the following situation. Eve cannot necessarily choose edges to be attacked by herself. However, she knows the positions of the attacked edges, and chooses her strategy depending on the attacked edges.

**Remark** **1.** *It is better to remark that there are two kinds of formulations in network coding, even when the network has only one sender and one receiver. Many papers [1,8,9,28,29] adopt the formulation, where the users can control the coding operation in intermediate nodes. However, this paper adopts another formulation, in which the non-local coding operations are done only for the input variable X and the output variable $Y_{B}$ like the papers [7,14,20,22,23,24]. In contrast, all intermediate nodes make only linear operations over a single transmission, which is often called local encoding in [22,23,24]. Since the linear operations in intermediate nodes cannot be controlled by the sender and the receiver, this formulation contains the case when a part of intermediate nodes do not work and output 0 always.*

*In the former setting, it is often allowed to employ the private randomness in intermediate nodes. However, we adopt the latter setting; i.e., no non-local coding operation is allowed in intermediate nodes, and each intermediate node is required to make the same linear operation on each alphabet. That is, the operations in intermediate nodes are linear and are not changed during n transmissions. The private randomness is not employed in intermediate nodes.*

Now, we have the following reduction theorem.

**Theorem** **2** (Reduction theorem)**.** *When the triplet $\left(K,H,\alpha^{n}\right)$ satisfies the uniqueness condition, Eve’s information $Y_{E}^{n}\left(\alpha^{n}\right)$ with strategy $\alpha^{n}$ can be calculated from Eve’s information $Y_{E}^{n}\left(0\right)$ with strategy 0 (the passive attack), and $Y_{E}^{n}\left(0\right)$ is also calculated from $Y_{E}^{n}\left(\alpha^{n}\right)$. Hence, we have the equation*

(27) $$\begin{array}{cc} I\left(M;Y_{E}^{n}\right)\left[\Phi_{n},K,0,0\right]=I\left(M;Y_{E}^{n}\right)\left[\Phi_{n},K,H,0\right]= & I\left(M;Y_{E}^{n}\right)\left[\Phi_{n},K,H,\alpha^{n}\right]. \end{array}$$

**Proof.**  Since the first equation follows from the definition, we show the second equation. We define two random variables $Y_{E}^{n}\left(0\right):=K_{E}X^{n}$ and $Y_{E}^{n}\left(\alpha^{n}\right):=K_{E}X^{n}+H_{E}Z^{n}$. Due to the uniqueness condition of $Y_{E}^{n}\left(\alpha^{n}\right)$, for each $Y_{E}^{n}\left(0\right)=K_{E}X^{n}$, we can uniquely identify $Y_{E}^{n}\left(\alpha^{n}\right)$. Therefore, we have $I\left(M;Y_{E}^{n}\left(0\right)\right)\geq I\left(M;Y_{E}^{n}\left(\alpha^{n}\right)\right)$. Conversely, since $Y_{E}^{n}\left(0\right)$ is given as a function of $Y_{E}^{n}\left(\alpha^{n}\right)$, $Z^{n}$, and $H_{E}$, we have the opposite inequality. □

**Remark** **2.** *Theorem 2 discusses the unicast case. It can be trivially extended to the multicast case because we do not discuss the decoder. It can also be extended to the multiple unicast case, whose network is composed of several pairs of senders and receivers. When there are k pairs in this setting, the messages M and the scramble random numbers L have the forms $\left(M_{1},\ldots ,M_{k}\right)$ and $\left(L_{1},\ldots ,L_{k}\right)$. Thus, we can apply Theorem 2 to the multiple unicast case. The detail discussion for this extension is discussed in the paper [33].*

**Remark** **3.** *One may consider the following type of attack when Alice sends the i-th transmission after Bob receives the i−1-th transmission. Eve changes the edge to be attacked in the i-th transmission dependently of the information that Eve obtains in the previous i−1 transmissions. Such an attack was discussed in [34]; there was no noise injection. Theorem 2 does not consider such a situation because it assumes that Eve attacks the same edges for each transmission. However, Theorem 2 can be applied to this kind of attack in the following way. That is, we find that Eve’s information with noise injection can be simulated by Eve’s information without noise injection even when the attacked edges are changed in the above way.*

*To see this reduction, we consider m transmissions over the network given by the direct graph (V,E). We define the big graph $\left(V_{m},E_{m}\right)$, where $V_{m}:={\left\{\left(v,i\right)\right\}}_{v\in V,1\leq i\leq m}$ and $E_{m}:={\left\{\left(e,i\right)\right\}}_{e\in E,1\leq i\leq m}$ and (v,i) and (e,i) express the vertex v and the edge e on the i-th transmission, respectively. Then, we can apply Theorem 2 with n=1 to the network given by the directed graph $\left(V_{m},E_{m}\right)$ when the attacked edges are changed in the above way. Hence, we obtain the above reduction statement under the uniqueness condition for the network decided by the directed graph $\left(V_{m},E_{m}\right)$.*

### 3.5. Application to Network Model in Section 2.6

We consider how to apply the multiple transmission setting with sequential transmission with n=2 to the network given in Section 2.6; i.e., we discuss the network given in Figure 1 and Figure 2 over the field $F_{q}$ with n=2. Then, we analyze the secrecy by applying Theorem 2.

Assume that Eve eavesdrops edges e(2),e(5),e(6),e(7),e(8) and contaminates edges e(2),e(5) as Figure 1. Then, we set the function $\tau_{E}$ from {1,…,11}×{1,2} to {1,…,22} as

(28) $$\begin{array}{c} \tau_{E}\left(i,l\right)=i+11\left(l-1\right). \end{array}$$

Under the choice of η given in (14), the function $\tau_{E,\eta }$ can be set in another way as

(29) $$\begin{array}{c} \tau_{E,\eta }\left(i,l\right)=i+2\left(l-1\right). \end{array}$$

Since $\gamma_{\bar{\theta }}\left(2,1\right)=7$, $\gamma_{\bar{\theta }}\left(5,1\right)=6$, $\gamma_{\bar{\theta }}\left(2,2\right)=18$, $\gamma_{\bar{\theta }}\left(5,2\right)=17$, we have

$$\begin{array}{cc} w_{\eta ,1,1}= & \left\{\left(1,1\right),\left(2,1\right)\right\},\;w_{\eta ,2,1}=\left\{\left(1,1\right),\left(2,1\right),\left(3,1\right)\right\}\\ w_{\eta ,1,2}= & \left\{(1,1),(2,1),(3,1),(4,1),(5,1),(1,2),(2,2)\right\}\\ w_{\eta ,2,2}= & \{(1,1),(2,1),(3,1),(4,1),(5,1),(1,2),(2,2),(3,2)\}. \end{array}$$

However, when the function $\tau_{E}$ is changed as

(30) $$\begin{array}{cc} \tau_{E}\left(i,l\right) & =i+5(l-1)\;\mathrm{for}\;i=1,\ldots ,5 \end{array}$$

(31) $$\begin{array}{cc} \tau_{E}\left(i,l\right) & =5+i+6(l-1)\;\mathrm{for}\;i=6,\ldots ,11, \end{array}$$

$w_{\eta ,i,l}$ has a different form as follows. Under the choice of η given in (14), while Eve can choose $\tau_{E,\eta }$ in the same way as (29), since $\gamma_{\bar{\theta }}\left(2,1\right)=12$, $\gamma_{\bar{\theta }}\left(5,1\right)=11$, $\gamma_{\bar{\theta }}\left(2,2\right)=18$, $\gamma_{\bar{\theta }}\left(5,2\right)=17$, we have

$$\begin{array}{cc} w_{\eta ,1,1}= & \left\{(1,1),(2,1),(1,2),(2,2)\right\}\\ w_{\eta ,2,1}= & \left\{(1,1),(2,1),(3,1),(1,2),(2,2)\right\}\\ w_{\eta ,1,2}= & \left\{(1,1),(2,1),(3,1),(4,1),(5,1),(1,2),(2,2)\right\}\\ w_{\eta ,2,2}= & \{(1,1),(2,1),(3,1),(4,1),(5,1),(1,2),(2,2),(3,2)\}. \end{array}$$

We construct a code, in which the secrecy holds and Bob can detect the existence of the error in this case. For this aim, we consider two cases: (i) there exists an element $\kappa \in F_{q}$ to satisfy the equation $\kappa^{2}=\kappa +1$; (ii) no element $\kappa \in F_{q}$ satisfies the equation $\kappa^{2}=\kappa +1$. Our code works even with n=1 in the case (i). However, it requires n=2 in the case (ii). For simplicity, we give our code with n=2 even in the case (i).

Assume the case (i). Alice’s message is $M=\left(M_{1},M_{2}\right)\in F_{q}^{2}$, and Alice prepares scramble random numbers $L_{i}=\left(L_{i,1},L_{i,2}\right)\in F_{q}^{2}$ with i=1,2. These variables are assumed to be subject to the uniform distribution independently. She encodes them as $X_{1}=L_{1}$, $X_{2}=L_{1}\kappa +L_{2}\left(1+\kappa \right)+M\kappa$, $X_{3}=L_{2}+M$, and $X_{4}=L_{2}$. When $Z_{1}=Z_{2}=0$, Bob receives

(32) $$\begin{array}{c} \begin{array}{cc} Y_{B,1} & =X_{1}+X_{2}=L_{1}\left(1+\kappa \right)+L_{2}\left(1+\kappa \right)+M\kappa ,\\ Y_{B,2} & =X_{1}+X_{3}=L_{1}+L_{2}+M,\\ Y_{B,3} & =X_{1}+X_{4}=L_{1}+L_{2}. \end{array} \end{array}$$

Then, since $M=Y_{B,2}-Y_{B,3}$, he recovers the message by using $Y_{B,2}-Y_{B,3}$.

As shown in the following, under this code, Eve cannot obtain any information for *M* even though she makes active attack under the model given Figure 1. Eve’s information is

(33) $$\begin{array}{c} \left(\begin{array}{c} Y_{E,1}\\ Y_{E,2}\\ Y_{E,3}\\ Y_{E,4}\\ Y_{E,5} \end{array}\right)=\left(\begin{array}{ccc} \kappa & \kappa & 1+\kappa \\ 0 & 1 & 0\\ 0 & 1 & 0\\ \kappa & 1+\kappa & 1+\kappa \\ 0 & 1 & 0 \end{array}\right)\left(\begin{array}{c} M\\ L_{1}\\ L_{2} \end{array}\right) \end{array}$$

when $Z_{i}=0$. Proposition 2 guarantees that Eve cannot obtain any information for *M* when $Z_{i}=0$. Thus, due to Theorem 2, the secrecy holds even when $Z_{i}=0$ does not hold.

Indeed, the above attack can be considered as the following. Eve can eavesdrop all edges connected to the intermediate node v(2) and contaminate all edges incoming to the intermediate node v(2). The above setting means that the intermediate node v(2) is partially captured by Eve. As other settings, we consider the case when Eve attacks another node v(i) for i=1,3,4. In this case, we allow a slightly stronger attack; i.e., Eve can eavesdrop and contaminate all edges connected to the intermediate node v(i). That is, Eve’s attack is summarized as

**(B1’)**  Eve can choose any one of nodes v(1),…,v(4). When v(2) is chosen, she eavesdrops all edges connected to v(2) and contaminates all edges incoming to v(2). When v(i) is chosen for i=1,3,4, she eavesdrops and contaminates all edges connected to v(i).

To apply Corollary 1 for analysis of the secrecy, we write down the low vectors $v_{i}$ in Corollary 1 under “Vector” in Table 2. Hence, under this attack, this code has the same secrecy by the combination of Corollary 1 and Theorem 2.

In the case (ii), we set κ as the matrix $\left(\begin{array}{cc} 0 & 1\\ 1 & 1 \end{array}\right)$. Then, we introduce the algebraic extension $F_{q}\left[\kappa \right]$ of the field $F_{q}$ by using the element *e* to satisfy the equation $\kappa^{2}=\kappa +1$. Then, we identify an element $\left(x_{1},x_{2}\right)\in F_{q}^{2}$ with $x_{1}+x_{2}\kappa \in F_{q}\left[\kappa \right]$. Hence, the multiplication of the matrix κ in $F_{q}^{2}$ can be identified with the multiplication of κ in $F_{q}\left[\kappa \right]$. The above analysis works by identifying $F_{q}^{2}$ with the algebraic extension $F_{q}\left[\kappa \right]$ in the case (ii).

### 3.6. Error Detection

Next, using the discussion in Section 2.2, we consider another type of security, i.e., the detectability of the existence of the error when n=2 with the assumptions (B1’), (B2) and the following alternative assumption:

**(B3’)**  The message set M is $F_{q}^{2}$, and all information on all edges per single use are $F_{q}$.
**(B4’)**  The encoder on the source node can be chosen, but is restricted to linear. It is allowed to use a scramble random number, which is an element of $L:=F_{q}^{k}$ with a certain integer *k*. Formally, the encoder is given as as a linear function from M×L to $F_{q}^{8}$.

We employ the code given in Section 3.5 and consider that the contamination exists when $Y_{B,1}-\left(Y_{B,3}+Y_{B,2}\kappa \right)$ is not zero. This code satisfies the secrecy and the detectability as follows.

To consider the case with v(2), we set η(1)=5,η(2)=2. Regardless of whether Eve makes contamination, $Y_{B,2}-Y_{B,3}=L_{1}+L_{2}+Z_{1}+M-\left(L_{1}+L_{2}+Z_{1}\right)=M$. In the following, $Y_{B,i}$ for i=1,2,3 expresses the variable when Eve makes contamination. Hence, Bob always recovers the original message *M*. Therefore, this code satisfies the desired security in the case with Figure 1.

In the case of v(3), we set η(1)=3,η(2)=6,η(3)=9. Then, $Y_{B,1}-\left(Y_{B,3}+Y_{B,2}\kappa \right)$ is calculated through $-(Z_{1}+Z_{2}+Z_{3})\kappa$. Hence, when $Z_{1}+Z_{2}+Z_{3}=0$, Bob detects no error. In this case, the contamination ($Z_{1}$, $Z_{2}$, and $Z_{3}$) does not change $Y_{B,2}-Y_{B,3}$, i.e., it does not cause any error for the decoded message. Hence, in order to detect an error in the decoded message, it is sufficient to check whether $Y_{B,1}-\left(Y_{B,3}+Y_{B,2}\kappa \right)$ is zero or not. Since $Y_{B,2}=X_{1}+X_{3}+Z_{1}+Z_{2}+Z_{3}$, we have $M\kappa =L_{1}\left(1+\kappa \right)+L_{2}\left(1+\kappa \right)+M\kappa -\left(L_{1}+L_{2}\right)\left(1+\kappa \right)=Y_{B,1}-Y_{B,3}\left(1+\kappa \right)$. Hence, if Bob knows that only the edges e(3), e(6), and e(9) are contaminated, he can recover the message by $\left(Y_{B,1}-Y_{B,3}\left(1+\kappa \right)\right)\kappa^{-1}$.

In the case of v(4), we set η(1)=4,η(2)=8,η(3)=10,η(4)=11. When $Y_{B,1}-\left(Y_{B,3}+Y_{B,2}\kappa \right)=-\left(Z_{1}+Z_{2}+Z_{4}\right)=0$, Bob detects no error. In this case, the errors $Z_{1}$, $Z_{2}$, and $Z_{4}$ do not change $Y_{B,2}-Y_{B,3}$. Hence, it is sufficient to check whether $Y_{B,1}-\left(Y_{B,3}+Y_{B,2}\kappa \right)$ is zero or not. In addition, if Bob knows that only the edges e(4),e(8),e(10),e(11) are contaminated, he can recover the message by $Y_{B,2}\left(1+\kappa \right)-Y_{B,1}$.

Similarly, in the case of v(1), we set η(1)=1, η(2)=5, η(3)=10. If Bob knows that only the edges e(1),e(5),e(10) are contaminated, he can recover the message by the original method $Y_{B,2}-Y_{B,3}$ because it equals $L_{1}+L_{2}+M+Z_{1}-\left(L_{1}+L_{2}+Z_{1}\right)$. In summary, when this type attack is done, Bob can detect the existence of the error. If he identifies the attacked node v(i) by another method, he can recover the message.

### 3.7. Solution of Problem Given in Section 2.7

Next, we consider how to resolve the problem arisen in Section 2.7. That is, we discuss another type of attack given as (B1), and study the secrecy and the detectability of the existence of the error under the above-explained code with the assumptions (B2), (B3’), (B4’), and (B5).

To discuss this problem, we divide this network into two layers. The lower layer consists of the edges e(7),e(9), and e(11), which are connected to the sink node. The upper layer does of the remaining edges. Eve eavesdrops and contaminates any one edge among the upper layer, and eavesdrops on any one edge among the lower layer.

Again, we consider the low vectors $v_{i}$ in Corollary 1. The vectors corresponding to the edges of the upper layer are (0,1,0), (κ,κ,1+κ), (1,0,1), and (0,0,1). The vectors corresponding to the edges of the lower layer are (κ,1+κ,1+κ), (1,1,1), and (0,1,1). Any linear combination from the upper and lower layers is not (1,0,0), which implies the secrecy condition given in Corollary 1. Hence, the secrecy holds under the lower type attack. Since the contamination of this type of attack is contained in the contamination of the attack discussed in the previous subsection, the detectability also holds.

## 4. Conclusions

We have discussed how sequential error injection affects the information leaked to Eve when node operations are linear. To discuss this problem, we have considered the possibility that the network does not have synchronization so that the information transmission on an edges starts before the end of the the information transmission on the previous edge. Hence, Eve might contaminate the information on several edges by using the original information of these edges. Additionally, we have discussed the multiple uses of the same network when the topology and the dynamics of the network do not change and there is no correlation with the delayed information.

As a result, we have shown that there is no advantage gained by injecting an artificial noise on attacked edges. This result can be regarded as a kind of reduction theorem because the secrecy analysis with contamination can be reduced to that without contamination. Indeed, when the linearity is not imposed, there is a counterexample of this reduction theorem [21].

In addition, we have derived the matrix formulas (20) and (21) for the relation between the outputs of Alice and Bob and the inputs of Alice and Eve in the case with the multiple transmission. As the extension of Theorem 1, the similar reduction theorem (Theorem 2) holds even for the multiple transmission. In fact, as explained in Section 3.7, this extension is essential because there exists an attack model over a network model such that the secrecy and the detectability of the error are possible with multiple uses of the same network, while it is impossible with the single use of the network. Additionally, another paper will discuss the application of these results to the asymptotic setting [33].

Indeed, there is a possibility that Eve changes $H_{E}$ sequentially. This problem has been done by the paper [35] essentially using the idea of our main theorems (Theorems 1 and 2) because it refers Proposition 1 of the conference version [36], which is equivalent to our main theorems. This fact shows the importance of our reduction theorem.

## Figures and Tables

**Table 1:** Channel parameters.

$m_{1}$	Number of edges
$m_{2}$	Number of vertecies
$m_{3}$	Dimension of Alice’s input information X
$m_{4}$	Dimension of Bob’s observed information $Y_{B}$
$m_{5}$	Dimension of Eve’s injected information Z
$m_{6}$	Dimension of Eve’s wiretapped information $Y_{E}$
$m_{7}$	$m_{1}-m_{3}$

**Table 2:** Summary of security analysis.

Node	Eavesdropping	Vector	η	Detection	Recovery
v(1)	e(1)	(0,1,0)	η(1)=1	$-Z_{1}\kappa$	$Y_{B,2}-Y_{B,3}$
	e(5)	(0,1,0)	η(2)=5		
	e(10)	(0,1,0)	η(3)=10		
v(2)	e(2)	(κ,κ,1+κ)		$Z_{2}-Z_{1}\kappa$	$Y_{B,2}-Y_{B,3}$
	e(5)	(0,1,0)	η(1)=5		
	e(6)	(0,1,0)			
	e(7)	(κ,1+κ,1+κ)	η(2)=2		
	e(8)	(0,1,0)			
v(3)	e(3)	(1,0,1)	η(1)=3	$-(Z_{1}+Z_{2}+Z_{3})\kappa$	$\left(Y_{B,1}-Y_{B,3}\left(1+\kappa \right)\right)\kappa^{-1}$
	e(6)	(0,1,0)	η(2)=6		
	e(9)	(1,1,1)	η(3)=9		
v(4)	e(4)	(0,0,1)	η(1)=4	$-Z_{1}-Z_{2}-Z_{4}$	$Y_{B,2}\left(1+\kappa \right)-Y_{B,1}$
	e(8)	(0,1,0)	η(2)=8		
	e(10)	(0,1,0)	η(3)=10		
	e(11)	(0,1,1)	η(4)=11		

Vector expresses the low vectors $v_{i}$ of the matrix *A* in Corollary 1. For the case with (2), the matrix *A* is given in Equation (33). Detection expresses $Y_{B,1}-\left(Y_{B,3}+Y_{B,2}\kappa \right)$. If this value is not zero, Bob considers that there exists the contamination. Recovery expresses Bob’s method that decodes the message *M* dependently of v(i).
